# Lessons from the implementation of LLIN distribution campaign in Ilorin Kwara State, Nigeria

**DOI:** 10.1186/1471-2458-14-514

**Published:** 2014-05-28

**Authors:** Abiodun Obembe, Okorie Okogbue Anyaele, Adedayo Olatunbosun Oduola

**Affiliations:** 1 Department of Biosciences and Biotechnology, Kwara State University Malete, Ilorin, Nigeria; 2 Entomology Unit, Department of Zoology, University of Ibadan, Ibadan, Nigeria; 3 Department of Zoology, University of Ilorin, Ilorin, Nigeria; 4 Molecular Entomology and Vector Control Research Laboratory, Nigerian Institute of Medical, Research, Yaba, Lagos, Nigeria

**Keywords:** Malaria, Long-lasting insecticidal nets, Sleeping habits, LLIN maintenance

## Abstract

**Background:**

Studies implemented to evaluate the success of Long-lasting insecticidal nets (LLIN) distribution campaigns are often limited to ownership and utilization rates, neglecting other factors that directly affect the efficacy of the tool in malaria control. This study investigates sleeping habits and net maintenance behaviour in addition to LLIN ownership, utilization and the challenges associated with LLIN use among residents in Ilorin City where the tool has been massively distributed.

**Methods:**

A cross-sectional survey was conducted using pre-tested interviewer-administered questionnaire to obtain information from randomly selected household respondents in Ilorin, the Kwara State Capital. The study was conducted in July 2012, about sixteen months after the March 2011 distribution of LLIN in the locality. The results were analyzed using the EPI INFO 2007 version.

**Results:**

LLIN ownership (85%) and utilization (37%) rates improved compared to earlier reports, though 29% of net users have noticed holes in the nets even as 26% claimed to have actually experienced mosquito bites under it. Most (92%) of the respondents who slept under LLIN the previous night before the study spent the first five hours of the night (19.00-23.00 hr) outdoors while 88% also engage in inappropriate net washing practices. All the LLIN users claimed to have experienced at least one malaria episode while 43% have had two or more episodes within the past twelve months.

**Conclusion:**

The use of LLIN among the respondents in this study was accompanied by chancy sleeping habits, inappropriate net maintenance practices and repeated experience of mosquito bites under the nets. This shows the need to sustain the will and confidence of LLIN users in this area through frequent monitoring and surveillance visits targeted at enlightening the people on habits that increase malaria exposure risks as well as proper use and maintenance of LLIN for maximum malaria vector control benefits.

## Background

Malaria vector control provides a preventive line of attack against the morbidity and mortality that may result from the disease. The World Health Organization (WHO) approaches to the control of malaria include vector control using Indoor Residual Spraying (IRS) and Long-lasting Insecticidal nets (LLIN). IRS though effective [1] requires on-site presence of unavailable skilled personnel to repeat the intervention after 6–12 months [2]. Nets on the other hand are effective, robust and easy to deliver vector control tools, and without them, it is unlikely that the goal of universal vector control coverage can be achieved and sustained in the most difficult-to-reach communities [3]. The use of treated nets has been known to reduce; numbers of infective mosquito bites by 70-90% [4], malaria morbidity by 50% [5], child mortality by 27% [6], incidence of the malaria parasite by 40% and malaria anaemia by nearly 50% [7].

Long-lasting insecticidal nets retain its effective biological activity without retreatment over a period of three years of recommended use under field conditions [8]. WHO position statement therefore holds that LLIN should be deployed beyond the level of achieving personal protection of a few most vulnerable to full coverage of all people at risk of malaria. The multiplied effects of several LLINs killing and reducing the population of vector mosquitoes in the community provide protection for all the residents including those who do not sleep under the nets [9,10]. Killeen *et. al*., [11] had also noted that modest coverage (around 60%) of all adults and children can achieve this equitable community-wide benefit.

These benefits of LLIN use can however be realized only when massive net distribution campaigns result in phenomenal increases in the rates of possession and appropriate utilization of the nets by the target population. In Nigeria, massive LLIN distribution campaign has been on since 2009, with few studies initiated to assess the ownership and utilization rates of the tool. However, none of the studies reporting LLIN ownership and utilization rates in Nigeria before or after massive net distribution campaigns [12-16], have considered other salient factors, such as sleeping habits and net maintenance behaviour, which can render ineffective the consistent use of the tool. It is noteworthy for instance that irregular washing practices leaves an accumulation of dust which reduces the efficacy of the nets against the vector mosquitoes just as people who usually stay outdoors for some night hours may have been exposed to infective malaria mosquito bites during the latter part of this period before sleeping under the nets. Moreover, since the massive distribution of LLIN in Ilorin, in March 2011, the impact of the distribution campaign on net ownership and utilization has not been assessed. This study therefore reports sleeping habits and net maintenance behaviour in addition to LLIN ownership, utilization and the challenges associated with its use among residents in Ilorin, Kwara State, after mass distribution of the nets in the locality.

## Methods

### Study area

The Study was conducted in Ilorin, the Kwara State Capital. The City is located within longitude 4o 35’E and latitude 8o 30’ N, covering a land area of about 150 Km^2^[17] with an estimated population of 766, 000 people as at 2006 [18]. The climate is tropical with mean annual temperature, relative humidity and rainfall of 27°C, 76% and 1800 mm, respectively [19]. Ilorin City is not just large but also unique in that it combines three different Local Governments areas; Ilorin west, Ilorin South and Ilorin East. Interestingly, only some parts of these three Local Government areas are present within the Ilorin metropolis. Other parts, including the district headquarters, of some of these Local Governments are located outside Ilorin City. The parts of the three Local Governments located within the Ilorin metropolis are not separated by clear cut boundaries. This study therefore considered only the areas within the Ilorin metropolis as one block for the survey rather than comparing the local government areas represented within the metropolis.

### Study design and data collection

The study adopted a cross-sectional survey approach and was carried out in July 2012. Two hundred and eighty households within the Metropolis were surveyed, using simple random sampling techniques. The sample size was determined from the formula for surveys based on a simple random sample [20] using malaria incidence rate of 81% [21], confidence level of 95%, 5% margin of error and 10% non-response and/or invalid response rate. Two hundred and fifty questionnaires were finally selected for analysis giving a non-response rate of 10.8%. The data collection instrument employed consists of a structured questionnaire which was self-administered by a team of trained interviewers. The questions contained therein covered socio-demographic characteristics, LLIN ownership and utilization, source of LLIN acquired, frequency of malaria occurrence since the beginning of net utilization, numbers of LLIN owned, coverage of freely distributed Government LLINs, knowledge of where to obtain LLIN in the areas as well as sleeping habits and net care practices.

### Data analysis

Data entry and analysis were done using EPI info 2007. The results were expressed as percentages while Chi-square test was used to determine the statistical significance of key observations and differences seen in cross tabulated variables. Level of statistical significance was set at *P* < 0.05.

### Ethical consideration

This study was approved by the Nigerian Institute of Medical Research Institutional Review Board. Verbal informed consent was obtained from each household head who also served as the respondent for the study. In situations where such heads were not available, informed consent was requested from a responsible adult representative who also served as the respondent.

## Results

### Socio-demographic profile of respondents

The total number of people in the 250 households represented was 1,645 giving an average household size of 6.58 people (Table 1). One hundred and fifty five (155) males and ninety five (95) females made up the total number (250) of respondents considered. Majority of the respondents (63%) were between the age group of 30 years and above compared with the 37% who were within the age range of 18–29 years. The numbers of respondents who have acquired tertiary education (200) were higher compared to secondary school leavers (43), primary school certificate holders (2) and those with no formal education (5). Most of the respondents (71%) were engaged in formal jobs (Civil servants), some were traders (14%), others artisans (9%) while a few others were either entrepreneurs (4%) or farmers (2%). Religious denomination of the respondents showed that 60% were Muslims as against 40% who were Christians.

**Table 1:** Socio-demographic profile of respondents

**Characteristics**	**Number (n = 250)**	**Percentage (%)**
**Sex**		
Male	155	62
Female	95	38
**Age**		
20-29	93	37
30 and above	157	63
**Educational status**		
No formal education	5	2
Primary	2	0.8
Secondary	43	17.2
Tertiary	200	80
**Occupation**		
Formal jobs	178	71
Traders	34	14
Artisans	23	9
Entrepreneurs	9	4
Farmers	6	2
**Religion**		
Muslims	151	60
Christians	99	40

n:total number of respondents considered.

### LLIN knowledge and coverage

The results obtained from questions bordering on knowledge of the respondents about LLIN, the extent of coverage of the net distribution campaign as well as ownership and source of LLIN showed that ninety-six percent of the respondents have heard about LLIN while the remaining 4% had not (Table 2). Knowledge of treated net was found to be insignificantly associated with the level of education (*P* = 0.413) and the type of occupation (*P* = 0.564) of the respondents. A total of 233 respondents were aware of the distribution of LLIN by the government compared to the remaining 17 who were unaware. One hundred and fifty eight respondents had no idea of where to get LLIN while the rest (92) knew where it could be purchased. The results on ownership of LLIN also show that 213 out of the 250 household respondents own LLIN while the remaining 37 did not. Knowledge of the LLIN was found to be significantly (*P* = 0.000) associated with its possession among the respondents. Eighty per cent of the 213 people who claimed to possess LLIN received it during the distribution campaign, 16% purchased it, and 4% bought some and were also given while only one person got it as a gift. Out of the 178 respondents who got the free government LLIN, 168 actually got it when it was been shared in their areas of residence while the remaining 10 respondents got the same government LLINs from friends and families who had received more than they needed. The other 82 respondents, apart from the 168, claimed that the free LLINs were neither brought to nor shared in their various areas of residence (Table 2). The areas of residence significantly (*P* = 0.026) affected whether the respondents got the free LLIN or not when likelihood ratio analysis was applied.

**Table 2:** Knowledge, ownership, coverage and source of LLIN among respondents

**Characteristics**	**Number (n = 250)**	**Percentages (%)**
**Knowledge of LLIN**		
Have heard of LLIN	241	96
Have never heard of LLIN	9	4
**Knowledge of LLIN distribution**		
Aware of net distribution	233	93
Not aware of net distribution	17	7
**Net distribution coverage**		
Nets were shared in our area	168	67
Nets were not shared in our area	82	33
**Ownership**		
LLIN owners	213	85
Non-owners	37	15
**Numbers of LLIN owned**		
None	37	15
One	56	22
Two	42	17
Three	40	16
More than three	75	30
**Source of LLIN**		
Government distribution	178	71
Bought	34	13.6
Gift from a politician	1	0.4
Non-owners	37	15
**Knowledge of where to buy LLIN**		
Know where to buy LLIN	92	37
Have no idea of where to buy LLIN	158	63

n:total number of respondents considered.

### Household LLIN utilization

As to the use of the LLINs, 192 out of the 213 people who had the nets claimed to have used it at least once while the remaining 21 were yet to use it (Table 3). The total number of people who had never used LLIN (those who owned it and those who did not) was 55. Five respondents out of the 192 that had used the LLIN at least once have completely discontinued its use while 94 respondents indicated that they do not use it at times. A total of 93 respondents slept under LLIN, the previous night before they were interviewed. This implies that the LLIN utilization rate among the 213 LLIN owners (93/213) and the whole of the 250 respondents (93/250) were 44% and 37% respectively (Table 3). The level of education (*P* = 0.096), sex (*P* = 0.569) and age group (*P* = 0.455) of the respondents were insignificantly associated with the use of LLIN the previous night before the study.

**Table 3:** Utilization indices of LLIN among the respondents

**Characteristics**	**Number**	**Percentage**
**Ever used LLIN**		
LLIN owners who have used it at least once	192	90
LLIN owners who have never used it	21	10
**Total**	**213**	**100**
**Frequency of LLIN use**		
LLIN owners who claimed to use it everyday	93	48
LLIN owners who do not use it everyday	94	49
Completely discontinued after the first use	5	3
**Total**	**192**	**100**
**LLIN use last night (among LLIN owners)**		
Owners of LLIN who did not use it last night	120	56
Owners of LLIN who used it last night	93	44
**Total**	**213**	**100**
**LLIN use last night (among all respondents)**		
Slept under LLIN	93	37
Did not Sleep under LLIN	157	63
**Total**	**250**	**100**

### Net care practices and sleeping habits among all respondents

The result of net care practices and sleeping habits among all the respondents have been summarized in Table 4. Twenty-six percent of the 187 respondents who still use the LLIN claimed to have been bitten by mosquitoes while sleeping under it while the remaining 74% indicated that they have never experienced such. Age group of the respondents did not significantly (*P* = 0.070) affect their encounter with mosquitoes while under LLIN. Most of the respondents (94%) indicated that they usually stay outdoors for some night hours (19.00 – 23.00 hr) before sleeping (indoors) while a few others (6%) did not. Eighty-five percent of the 235 respondents who stayed outdoors noted that they usually get bitten by mosquitoes while outdoors compared to the other 15% who had not noticed mosquito bites at such times. The sex of the respondents did not significantly (*P* = 0.193) affect their experience of mosquito bites while outdoors. Out of the 187 respondents who still use LLIN, 36% have never washed it, 16% do so monthly, 12% weekly, 10% fortnightly, 8% wash every two to three months, 8% twice a year, 4% wash it once a year while the remaining 12 respondents comprise another 4% who wash the nets once in 2 years, only one person thrice a month, two people who were not specific and one respondent that purchases another one when the old one gets dirty (Table 4).

**Table 4:** Net care practices and sleeping habits among all respondents

**Characteristics**	**Number**	**Percentage (%)**
**Net washing practices**		
Never washed LLIN	68	36
Wash LLIN monthly	30	16
Weekly	22	12
Fortnightly	18	10
Every 2–3 months	15	8
Every 6 months	14	8
Once a year	8	4
Others	12	6
**Total**	**187**	**100**
**Hole in nets**		
Have noticed hole in net	54	29
Have not noticed any hole in net	133	71
**Total**	**187**	**100**
**Mosquito bites under LLIN**		
Experienced mosquito bites while under LLIN	48	26
Have never experienced such	139	74
**Total**	**187**	**100**
**Sleeping habits**		
Stay outdoors between 19.00-23.00 hr	235	94
Do not	15	6
**Total**	**250**	**100**
**Outdoor mosquito bites**		
Often experience mosquito bites while outdoors	199	85
Do not experience bites while outdoors	36	15
**Total**	**235**	**100**

### Nature of discomfort experienced by LLIN users

**Figure 1:**
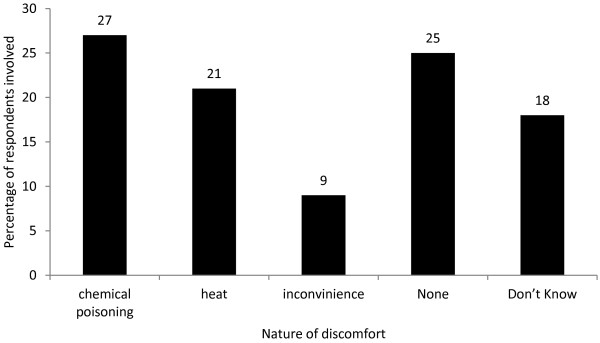
Nature of discomfort experienced by LLIN users.

More than a quarter (51) of all the 192 respondents who had used the LLIN at least once complained of chemical poisoning leading to adverse reactions such as swelling of the face, peppering of the eyes and hand pilling while 48 others claimed that they have not experienced any problem so far (Figure 1). Some respondents (34) who might have long discontinued LLIN use noted that they have no idea of the problems associated with it while others (18) experienced inconveniences in terms of the time to tuck and remove the net daily, difficulty in waking up at night and the inability to spread the body and turn from side to side.

### Sleeping habit, net care practices and malaria episodes among LLIN users

Most (86) of the 93 respondents who claimed to use the nets the previous night stay outdoors between 19.00-23.00 hr. Twenty seven percent of these LLIN users had noticed holes in the nets while some (19%) others claimed to have experienced mosquito bites under it. All the 93 respondents claimed to have experienced at least one malaria episode within the past 12 months while 43% claimed to have had two or more episodes (Table 5). The number of people who have experienced malaria episodes among users of LLIN last night before the study was not significantly (*P* = 0.329) different from the non-users. Nearly half (43%) of these respondents who claimed to have had malaria episodes despite consistent LLIN use wash their nets too frequently (1–4 times per month), 9% wash every six months, 3% were not specific while 32% comprised those who have never washed (20%) or have done so only once (13%) since the nets were acquired. Overall, this gives a total of 88% of respondents among the LLIN users involved in inappropriate net washing practices.

**Table 5:** Sleeping habit, net care practices and malaria episodes among LLIN users

**Characteristics**	**Number**	**Percentage**
	**(n = 93)**	**(%)**
**Sleeping habits**		
stay outdoors between 19.00-23.00 hr	86	92
Do not stay outdoors	7	8
**Net washing practices**		
Weekly	9	10
Every 2 weeks	8	9
Monthly	23	24
Every 2–3 months	11	12
Every 6 months	8	9
Only once since acquired	12	13
Not specific (when it is dirty)	3	3
Have never washed since acquired	19	20
**Hole in the nets**		
Net have hole(s)	25	27
Net does not have hole(s)	68	73
**Mosquito bites under treated net**		
Have experience bites while under treated net	18	19
Have not experienced such	75	81
**Malaria episodes in the past 12 months**		
Experienced it only once	53	57
Had malaria 2–4 times	32	34
Had malaria more than 4 times	8	9

n:number of respondents who used their LLINs the previous night.

## Discussion

The average household size obtained here was higher than the 5.7 persons reported by the National Population Commission (NPC) [22] as the average in Nigeria. Similar average household size to the 6.58 observed in this study has been reported in other Northern parts of Nigeria such as North West 6.5 [14] and North East regions 6.5 [23] as compared to those of South East 4.62 [13], South South 5.0 [23] and Southwest 4.7 [14]. There were many more males than female respondents in this study because the targets from the outset were the household heads. The high level of literacy recorded among the respondents in this study is typical of South-western states like Oyo, Osun and Ekiti states with which the people of Ilorin (North central) share borders and common language. Yahaya and Abubakar [24] had noted that Ilorin and its people serve as the melting point between the Northern and Southern cultures. Ilorin is one of the few towns in Nigeria with six tertiary institutions apart from the privately owned ones. Omokanye *et. al*., [25] had reported that most (60.3%) of the pregnant women attending antenatal clinic at the Ilorin University teaching hospital had tertiary education. This is in contrast with the low percentage (5%) of people with tertiary education in Sahel Savannah Northern States [23].

The mass distribution of free LLIN in this locality more than one year before the study must have accounted for the widespread knowledge (96%) and ownership (85%) as well as the 37% utilization rates of LLIN among the respondents as against low rates evident in other studies [12-14,26] conducted prior to such massive net distribution campaigns. A post-net distribution campaign study conducted in Kano (North West Nigeria) had reported an increase in LLIN ownership rate from 6% to 71% [16] while another study had also noted increased LLIN utilization rates from 47% to 89% in Plateau State (North Central) [15].

Most (83%) of the 213 households who owned LLIN in this study got it through the mass distribution of the nets in their areas of residence. However, the percentage of respondents (33%) who did not get these free LLINs as well as those (89%) who got more than two show that there were lapses in the distribution network. Besides, the considerable percentage (10%) of people who had never used their free LLINs, those (26%) who claimed to have been bitten by mosquitoes while under the nets, persons (36%) who have never washed their nets and the several complains of adverse reactions such as swelling of the face, peppering of the eyes and hand pilling suggest that a number of the respondents might be unaware of critical issues like drying the nets for 24 hrs before the first use and the proper way to tuck it under the bed or mat. The adverse reactions experienced have critical implications especially in terms of reducing the acceptability of LLIN as a vector control tool in this region. The reason for such reactions should be clearly explained to residents of this metropolis in order to allay their fears and correct any existing misconceptions. Also, the percentage of respondents (63%) who do not know where to obtain LLIN within the metropolis was higher than the 29.6% reported earlier in Ilorin [25] probably because the latter was conducted among pregnant women attending Antenatal clinic at the University teaching hospital. Again this shows that the social marketing strategy which is supposed to accompany this free LLIN distribution is either ineffective or not on course. This will negatively impact the fair outcome recorded so far especially if interested residents could not get LLIN easily from their areas to replace the ones they currently have when it expires.

The results also show that most (88%) of the LLIN users in this study engage in inappropriate net washing practices as against the twenty approved WHO washes during the three year life span of the net [8]. Keeping in mind that the present LLIN in this study area were distributed sixteen months before this study was conducted, it becomes evidenced why there were claims of holes in the nets. Therefore apart from the fact that the respondents may have failed to tuck in their nets properly, inappropriate net washing practices, which reduces the amount of active insecticide on the net [27], may have resulted in the easy passage of the vectors to the net occupants through available holes. This report is in consonance with observations of holes in LLINs [27-30] after some time while the access of mosquitoes to net occupants through small holes has also been reported [31].

The observation that 94% of all the respondents in this study were exposed out of which 85% experience mosquito bites while still outdoors corroborates earlier reports [32,33] of considerable *Anopheles* mosquito biting activity around this time in various Nigerian localities. Therefore the outdoor staying habits of many households may have resulted into the establishment of human-vector contact leading to the recorded malaria incidence claim by some respondents. The possible reasons for this outdoor staying behaviour, in urban settings like the study area, may be as a result of unstable electricity supply coupled with excessive heat experienced at certain seasons in this region. Policy makers should note that the provision of basic amenities like power supply would go a long way in improving the utilization rates of LLIN for malaria control in the worse hit African region.

While the sincerity of the respondents regarding every day use of LLIN remains an issue on one hand, the discouragement due to frequent mosquito bites under the nets and the claim of recurrent experience of malaria episodes is a serious concern on the other hand. Bearing in mind that the success of user-dependent tools like LLIN is determined by the continued will of the target population to put it to consistent use, the result of this study therefore bring to fore the need for frequent monitoring and surveillance in order to sustain the confidence of the users. Such surveillance and monitoring visits should focus on enlightening the people on the proper use and maintenance of their nets in order to achieve maximum malaria vector control benefits.

There is also the need to ascertain LLIN efficacy against mosquitoes over time considering the fact that unacceptably low efficacy of LLIN after only six washes has been reported in some African countries [27]. Moreover, efforts should be made to replace these twenty seven months old LLINs (as at May 2014) since about 40% according to Shirayama *et. al*. [30] or two-third according to Haji *et. al*. [27] of such nets could already be damaged after only two to three years of field use depending on the type of bed structure and where they are being used. Direct observations of LLIN in a state that ascertain its use the last nights before the interviews were not conducted in this study just as incidence of malaria parasite were also not established. These limitations should be considered and addressed in future investigations.

## Conclusion

LLIN use was accompanied by chancy sleeping habits, inappropriate net maintenance practices and repeated experience of mosquito bites under the nets among the respondents. This shows the need to sustain the will and confidence of LLIN users in this area through frequent monitoring and surveillance visits targeted at enlightening the people on out of harm's way sleeping habits as well as proper use and maintenance of their nets for maximum malaria vector control benefits. Stakeholders involved in behavioural change communication during and after massive LLIN distribution should also note these aspects in their campaigns with emphasis on pregnant women and children under the age of five.

## Abbreviations

WHO: World health organization; LLIN: Long-lasting Insecticidal Nets; IRS: Indoor residual spraying; NPC: National population commission.

## Competing interests

The authors declare that they have no competing interests.

## Authors’ contributions

AO and OOA conceived of the study and coordinated the team of interviewers on the field. OOA and AOO participated in the study design. AO drafted the manuscript. OOA and AOO helped in editing the overall manuscript. All authors read and approved the final manuscript.

## Pre-publication history

The pre-publication history for this paper can be accessed here:

http://www.biomedcentral.com/1471-2458/14/514/prepub
